# Nutrition Can Modulate the Toxicity of Environmental Pollutants: Implications in Risk Assessment and Human Health

**DOI:** 10.1289/ehp.1104712

**Published:** 2012-02-22

**Authors:** Bernhard Hennig, Lindell Ormsbee, Craig J. McClain, Bruce A. Watkins, Bruce Blumberg, Leonidas G. Bachas, Wayne Sanderson, Claudia Thompson, William A. Suk

**Affiliations:** 1 University of Kentucky Superfund Research Program, Lexington, Kentucky, USA; 2 University of Louisville School of Medicine, Louisville, Kentucky, USA; 3 Department of Nutritional Sciences, University of Connecticut, Storrs, Connecticut, USA; 4 School of Biological Sciences, University of California–Irvine, Irvine, California, USA; 5 Department of Chemistry, University of Miami, Coral Gables, Florida, USA; 6 Department of Epidemiology, University of Kentucky, Lexington, Kentucky, USA; 7 Susceptibility and Population Health, and; 8 Center for Risk and Integrated Sciences, National Institute of Environmental Health Sciences, National Institutes of Health, Department of Health and Human Services, Research Triangle Park, North Carolina, USA

**Keywords:** anti-inflammatory nutrients, environmental pollutants, nutrition, risk assessment, risk reduction

## Abstract

Background: The paradigm of human risk assessment includes many variables that must be viewed collectively in order to improve human health and prevent chronic disease. The pathology of chronic diseases is complex, however, and may be influenced by exposure to environmental pollu-tants, a sedentary lifestyle, and poor dietary habits. Much of the emerging evidence suggests that nutrition can modulate the toxicity of environmental pollutants, which may alter human risks associated with toxicant exposures.

Objectives: In this commentary, we discuss the basis for recommending that nutrition be considered a critical variable in disease outcomes associated with exposure to environmental pollutants, thus establishing the importance of incorporating nutrition within the context of cumulative risk assessment.

Discussion: A convincing body of research indicates that nutrition is a modulator of vulnerability to environmental insults; thus, it is timely to consider nutrition as a vital component of human risk assessment. Nutrition may serve as either an agonist or an antagonist (e.g., high-fat foods or foods rich in antioxidants, respectively) of the health impacts associated with exposure to environmental pollutants. Dietary practices and food choices may help explain the large variability observed in human risk assessment.

Conclusion: We recommend that nutrition and dietary practices be incorporated into future environmental research and the development of risk assessment paradigms. Healthful nutrition interventions might be a powerful approach to reduce disease risks associated with many environmental toxic insults and should be considered a variable within the context of cumulative risk assessment and, where appropriate, a potential tool for subsequent risk reduction.

The U.S. Environmental Protection Agency (EPA) defines risk as “the chance of harmful effects to human health or to ecological systems resulting from exposure to an environmental stressor” (U.S. EPA 2011). As the use of chemicals and pollutant emissions increase (i.e., as more chemicals or toxic substances are used in manufacturing and other venues, such as agriculture), it has become clear that humans are environmentally exposed not only to an ever increasing number of potential toxicants but also to harmful mixtures of these toxic substances. As a result, the U.S. EPA began addressing the issue of cumulative risk assessment with its *Framework for Cumulative Risk Assessment*, a report that defines cumulative risk as the combined risks to health by multiple agents or stressors (U.S. EPA 2003). In addition, a report by the National Research Council (2009), strongly recommended nonchemical stressors (psychosocial, physical, and dietary variables) in risk assessment, even in the absence of population-specific data. These cumulative risk debates and recent publications (Lewis et al. 2011; National Research Council 2009; Sexton and Linder 2011) substantially support our hypothe-sis that unhealthy dietary practices by themselves can compromise health, thus further increasing a person’s vulnerability to additional chemical stressors. In contrast, intervention with healthy dietary practices can contribute to health and metabolic stability, thus potentially reducing vulnerability to -disease-causing environmental pollutants.

Diet-related chronic diseases represent the single largest cause of morbidity and mortality worldwide, and the health burden of obesity-related disease complications and of other chronic health problems continues to grow (Astrup et al. 2008; Satia 2009). In fact, the United Nations High-Level Meeting on Non-communicable Diseases, held in September 2011, highlighted the urgent need to address the 57 million deaths per year attributed to noncommunicable diseases (of which cardiovascular disease plays a primary role) through an increased focus on research and development, a better understanding of the factors leading to these diseases, and more effective research translation (United Nations General Assembly 2011). The World Health Organization has noted that unhealthy diets and physical inactivity are key risk factors for the three primary non-communicable diseases (cardiovascular diseases, cancer, and diabetes). To address these growing concerns, in 2004 the World Health Assembly adopted the World Health Organization Global Strategy on Diet, Physical Activity and Health, which provides a strategy for the wide-scale promotion of healthy diets and increased physical activity (World Health Organization 2004).

Significant and relatively recent changes in dietary habits and other lifestyle conditions, for example, the introduction of processed foods and the sedentary nature of modern transportation, have been too rapid for the human genome to adjust, and it is believed that such unhealthy changes in nutritional, cultural, and activity patterns may underlie many of the “chronic diseases of Western civilization” (Cordain et al. 2005). In contrast, most chronic diseases, including coronary heart disease and type 2 diabetes, can be avoided or attenuated by healthy dietary lifestyles that are consistent with the traditional Mediterranean diet (Kastorini and Panagiotakos 2009; Sofi 2009; Willett 2006). This diet includes high ratios of mono-unsaturated to saturated fats and omega-3 to omega-6 fatty acids, plus an ample supply of fruits, vegetables, legumes, and whole grains (Galland 2010)—that is, foods rich in antioxidant and anti-inflammatory nutrients.

The complex interplay of nutrition and environmental pollutants in disease risk is influenced not only by nutrients that can modulate environmental insults but also by food that can serve as a source of healthy nutrients as well as contaminants. Furthermore, recent findings indicate that obesogens, in addition to a diet-induced positive energy balance, may contribute to obesity and associated health problems (Grün and Blumberg 2009b; Holtcamp 2012). Obesogens are environ-mental chemicals that might synergize with specific nutrients to accelerate metabolic dysfunctions or to interfere with mechanisms that regulate adipocyte number and energy balance, thus leading to compromised health associated with obesity (Janesick and Blumberg 2011).

To explore further the paradigm that nutrition can modulate toxicological insults, and to identify the potential implications of this paradigm for risk assessment of environmental pollutants and human health, the University of Kentucky Superfund Research Center invited experts from the fields of nutritional sciences, medicine, public health, and environmental toxicology, as well as scientists from the U.S. EPA National Center for Environmental Assessment, to participate in a workshop titled “Nutrition and Chemical Toxicity: Implications in Risk Assessment.” In a previous commentary (Hennig et al. 2007), we stated how nutrition can be used to modulate toxicological events associated with exposure to hazardous substances. Here we expand on this reasoning, and on a growing body of evidence supporting its validity, to explore new ways of incorporating nutrition within the paradigm of risk assessment. Thus, this recent meeting focused on nutrition as a modulator of environ-mental toxicity and on the need to consider nutrition (diet) as a component of risk assessment methodologies. Presentations highlighted studies that suggest that nutrition can be a potential modulator of diseases associated with exposure to environ-mental stressors. However, critical questions remain. For example, to what extent can health risks associated with exposure to environ-mental pollutants be reduced or improved through healthy nutrition? Can nutrition be a critical component in redefining methodologies used in risk assessment? That is, can nutrition (or dietary practices) be considered either a stressor or a buffer of cumulative risk from exposure to environmental pollutants?

## Discussion

Healthy nutrition can positively influence, or lessen, the human health risks associated with exposure to mixtures of environmental chemi-cals (Hennig et al. 2007). As some of the authors have previously asserted, “Nutritional interventions may provide the most sensible means to develop primary prevention strategies for diseases associated with many environ-mental toxic insults” (Hennig et al. 2007). Building upon the hypothesis that nutrition is a modulator of environmental toxicity, and considering the mounting evidence in support of this hypothesis, this paradigm needs to be considered now at the level of risk assessment.

Many persistent environmental pollutants, including polychlorinated biphenyls (PCBs), brominated flame retardants, and organometallic compounds, can accumulate in the body. In addition, these persistent organic pollutants can generate free radicals, which in turn can trigger proinflammatory signaling pathways and associated inflammatory diseases, including atherosclerosis, diabetes, and hypertension (Ha et al. 2007; Hectors et al. 2011; Sergeev and Carpenter 2011). B.H.’s laboratory has conducted extensive research over the years to study the effects of PCBs on the early pathology of atherosclerosis, with a particular focus on vascular endothelial cell function. These studies have shown that an increase in cellular oxidative stress and an imbalance in antioxidant status are critical events in PCB-mediated induction of inflammatory genes and endothelial cell dysfunction (reviewed by Hennig et al. 2005). The research team also found that specific dietary fats can further compromise endothelial dysfunction induced by selected PCBs by further adding to a cellular oxidative and inflammatory insult. Importantly, study data suggest that antioxidant nutrients, such as vitamin E and dietary flavonoids, as well as a high ratio of omega-3 to omega-6 fatty acids, can protect against endothelial cell damage mediated by these persistent organic pollutants (Hennig et al. 2005; Majkova et al. 2011; Wang et al. 2008). Recent data further suggest that membrane lipid rafts such as caveolae may play a major role in the regulation of PCB-induced inflammatory signaling in endothelial cells, as well as in protective mechanisms of dietary-derived polyphenols such as quercetin and the green tea catechin epigallocatechin-gallate (Majkova et al. 2010; Zheng et al. 2012). These studies provide strong evidence that healthy nutrition can protect against proinflammatory environmental stressors. However, human studies are needed to confirm such therapeutic effects of protective nutrients. In the same context, it is important to be mindful that long-term effects of excessive antioxidant intake in the form of supplements have been questioned (Gaziano et al. 2009; Soni et al. 2010). In addition, a multitude of bioactive food components occur in the food supply, and some of these bioactive constituents likely share the same molecular targets. Thus, continued research and a greater understanding of nutrition science will be needed to optimize any dietary intervention in order to reduce health risks associated with exposure to environmental pollutants. Evolving studies suggest that the interaction of toxicants and nutrients occurs throughout the body, and that the consequences of tissue damage by environmental pollutants can be significant. For example, a review of the National Health and Nutrition Examination Survey (NHANES) found that PCB, lead, and mercury exposures were associated with elevated blood levels of alanine aminotransferase (ALT), a marker for nonalcoholic fatty liver disease (Cave et al. 2010a). Interestingly, high-fructose diets also can induce non-alcoholic fatty livers or steatohepatitis, which was accompanied by a multi-fold increase in blood ALT levels (Kanuri et al. 2010). Nonalcoholic steatohepatitis is usually associated with obesity, but it also has been reported in lean individuals exposed to industrial chemicals, such as vinyl chloride (Cave et al. 2010b). Taken together, these studies suggest that the involvement of similar and possibly additive liver pathologies can be induced by both environmental pollutants and certain dietary components.

Increasing evidence indicates that anti-inflammatory nutrients, in particular dietary long-chain omega-3 polyunsaturated fatty acids and plant-derived flavonoids, can be used as a therapeutic approach to deter toxico-logical insults and pathologies associated with exposure to environmental pollutants (Watkins et al. 2007). Of interest is the fact that dietary lipids rich in omega-6 fatty acids, including linoleic and arachidonic acids, can amplify inflammatory outcomes of persistent organic pollutants (Arzuaga et al. 2009). In contrast, changing the ratios of omega-6 to omega-3 fatty acids can differentially modulate PCB-induced inflammatory parameters in vascular endothelial cells (Wang et al. 2008). It appears that fish-derived omega-3 fatty acids are extremely protective against pollutant-induced inflammation, and membrane domains such as caveolae or other types of lipid rafts may provide a regulatory platform for cellular protection by omega-3 fatty acids (Layne et al. 2011; Majkova et al. 2010).

Endocannabinoid signaling can be a target for cellular insults by both environmental pollutants and long-chain omega-6 fatty acids (e.g., arachidonic acid) (Watkins et al. 2010). Endocannabinoids are lipid or lipophilic mediators that bind to specific receptors and elicit cell signaling. Novel mechanisms involving the endocannabinoid signaling pathway have been implicated as important in exacerbating the effects of environmental pollutants. Recent findings suggest that an imbalance of endocannabinoid signaling can contribute to visceral fat accumulation and disruption of energy homeostasis, which are characteristics of metabolic syndrome (Kim et al. 2011). Interestingly, dietary omega-3 polyunsaturated fatty acids can reverse the dysregulation of the endocannabinoid system, which is linked to a decrease in insulin resistance and body fat (Kim et al. 2011). This may explain observations that certain PCBs can promote obesity and atherosclerosis (Arsenescu et al. 2008), as well as how omega-3 poly-unsaturated fatty acids can reduce body fat deposition and improve obesity-induced metabolic syndrome (Tai and Ding 2010).

Another example of the complex interplay of diet and environmental pollutants is the obesogen model, which explains in part the current proliferation of obesity-associated diseases. Although obesity generally occurs when energy intake exceeds energy expenditure, individual accumulation of fat mass under similar energy intake can vary widely not only because of genetic predispositions but possibly also because of exposure to environmental pollutants. The environmental obesogen hypothesis is based on the assumption that prenatal or early-life exposure to certain endocrine-disrupting chemicals can interfere with the body’s adipose tissue homeostasis and the ability to regulate weight control, thus leading to increased fat mass and obesity in exposed individuals later in life (Grün and Blumberg 2009a). For example, prenatal exposure to tributyltin chloride can cause inappropriate activation of retinoid X receptor–peroxisome proliferator-activated receptor signaling, thus modulating adipogenic genes and leading to increased adiposity and obesity (Grün and Blumberg 2006).

There is strong evidence that exposure to environmental pollutants, especially persistent organic pollutants, provides added risk for the development of many diseases. Causative and mechanistic data of certain pathologies have emerged mostly from animal studies, but cross-sectional human studies also provide evidence that humans who live near hazardous waste sites are more prone to exhibit diseases associated with high blood pressure, cardio-vascular dysfunction, and insulin resistance (Goncharov et al. 2011; Sergeev and Carpenter 2005). In addition, diet-related chronic diseases are now considered the largest cause of morbidity and mortality worldwide (Astrup et al. 2008). It is clear that human health assessment methodologies need to consider the complexity of multiple stressors to understand the cumulative risk to human health from exposure to environmental pollutants. The U.S. EPA is advocating for new risk assessment paradigms, such as community-based risk assessment, that aim to address the cumulative stressors faced by a particular community (Sanchez et al. 2010). These new concepts of risk assessment assume varied physio-logical outcomes related to exposure to mixtures of stressors (chemical and non-chemical) in assessing health risks to biological compartments and, ultimately, to the whole body.

Although nutritional interventions (e.g., anti-oxidants) have been shown to be of benefit in animal studies, the robustness of the results is sometimes diminished in human inter-vention studies (Soni et al. 2010). However, this fact does not invalidate the overall benefit of healthy nutrition; more research is needed to understand the human variability associated with such results. It is recognized that for a given individual the impact of a particular nutrient will vary with the amount consumed. Such variability is influenced by multiple factors including an individual’s genome (e.g., polymorphisms). Thus, genomics should not be overlooked in public health policy (Fenech et al. 2011; Gerard et al. 2002). Furthermore, an important but largely unexplored issue is the combined epigenetic effects of nutrition and pollutants and their implications in risk assessment and human health. Future research in nutrigenetics and nutrigenomics is likely to yield individualized nutritional inter-vention plans that will allow risk assessments to directly incorporate such variability. Given this probability, it is important that we begin to consider the possible implications of such developments in the context of risk assessment and nutritional intervention.

## Conclusions and Recommendations

**Figure 1:**
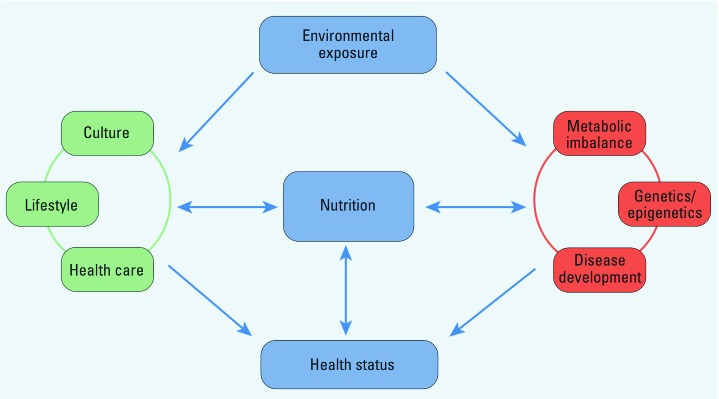
Illustration of nutrition as a modulator in the interplay of health status associated with exposure to environmental pollutants. In the traditional context of risk assessment, risk is associated with the “chance of harmful effects to human health resulting from exposure to an environmental stressor” (U.S. EPA 2011). We propose that nutrition can modulate this paradigm throughout one’s life span and that lifestyle choices that include healthy nutrition can reduce health risks associated with hazardous substances, which clearly fits within the paradigm of risk assessment.

Multiple stressors could synergize risk related to exposure to an individual toxicant or a mixture of pollutants; in contrast, understanding and attempting to eliminate non-chemical stressors within the context of cumulative risk assessment could reduce the risk from chemical stressors or pollutants. Thus, the issue of cumulative risk to human health should include overall health outcomes associated with diet or nutrition, as well as other lifestyle choices (Figure 1). Nutrition or dietary choices can modulate chemical insults associated with exposure to environmental pollutants. This is especially important because nutrition can both exacerbate and attenuate many disease indicators, such as oxidative stress and inflammation, that are linked to health risks associated with exposures (Hennig et al. 2007). Furthermore, exposure to environmental pollutants can be chronic throughout life, thus providing opportunities for toxic chemicals to contribute to disease development beginning in childhood and continuing into adult life (Gilliland et al. 2005; Landrigan et al. 2005; Wilson and Suk 2010). It is plausible that individuals who are compromised nutritionally (e.g., because of poor dietary habits) are more vulnerable to hazardous chemicals throughout their life span. In contrast, diets rich in anti-oxidants and anti-inflammatory nutrients can improve health and decrease vulnerability to additional chemical stressors. Thus, healthy nutrition intervention should be considered as early in life as possible. We recommend that nutrition be included as a critical variable in risk assessment methodologies, and that this incorporation should emphasize nutrition as a powerful intervention tool for lowering health risks related to environ-mental toxicants. In fact, healthful nutrition could markedly buffer the body against chemical, biological, and physi-cal stressors that humans are exposed to on a daily basis. Thus, positive dietary behaviors can potentially reduce health risks associated with hazardous substances, clearly placing nutrition within the paradigm of risk assessment. Healthful nutrition could also potentially lower significantly long-term remediation costs by protecting human health against uncertain risks linked to environmental pollutants.
